# Evaluation of reference genes for gene expression analysis by real-time quantitative PCR (qPCR) in three stingless bee species (Hymenoptera: Apidae: Meliponini)

**DOI:** 10.1038/s41598-019-53544-0

**Published:** 2019-11-27

**Authors:** Flávia C. P. Freitas, Thiago S. Depintor, Lucas T. Agostini, Danielle Luna-Lucena, Francis M. F. Nunes, Márcia M. G. Bitondi, Zilá L. P. Simões, Anete P. Lourenço

**Affiliations:** 1 Departamento de Genética, Faculdade de Medicina de Ribeirão Preto, Universidade de São Paulo, Ribeirão Preto, SP Brazil; 2 Instituto de Ciências Biomédicas, Universidade Federal de Alfenas, Alfenas, MG Brazil; 3 Departamento de Biologia, Faculdade de Filosofia, Ciências e Letras de Ribeirão Preto, Universidade de São Paulo, Ribeirão Preto, SP Brazil; 4 Departamento de Genética e Evolução, Centro de Ciências Biológicas e da Saúde, Universidade Federal de São Carlos, São Carlos, SP Brazil; 5 Departamento de Ciências Biológicas, Universidade Federal dos Vales do Jequitinhonha e Mucuri, Diamantina, MG Brazil

**Keywords:** Gene expression analysis, Transcription

## Abstract

Stingless bees are generalist pollinators distributed through the pantropical region. There is growing evidence that their wild populations are experiencing substantial decline in response to habitat degradation and pesticides. Policies for conservation of endangered species will benefit from studies focusing on genetic and molecular aspects of their development and behavior. The most common method for looking at gene expression is real-time quantitative polymerase chain reaction preceded by reverse transcription (RT-qPCR) of the mRNA of interest. This method requires the identification of reliable reference genes to correctly estimate fluctuations in transcript levels. To contribute to molecular studies on stingless bees, we used *Frieseomelitta varia*, *Melipona quadrifasciata*, and *Scaptotrigona bipunctata* species to test the expression stability of eight reference genes (*act*, *ef1-α*, *gapdh*, *rpl32*, *rps5*, *rps18*, *tbp*, and *tbp-af*) in RT-qPCR procedures in five physiological and experimental conditions (development, sex, tissues, bacteria injection, and pesticide exposure). In general, the *rpl32*, *rps5* and *rps18* ribosomal protein genes and *tpb-af* gene showed the highest stability, thus being identified as suitable reference genes for the three stingless bee species and defined conditions. Our results also emphasized the need to evaluate the stability of candidate genes for any designed experimental condition and stingless bee species.

## Introduction

A global effort to sequence genomes of all living species on Earth is in progress^1^ with the aims of mitigating the impact of climate changes on biodiversity and preserving endangered species and ecosystems. In particular, the i5K consortium leads the arthropod genomics initiatives and emphasizes on sequencing insect genomes^2^. The expanded availability of genomic data during the last decade created a favorable condition for researchers to investigate a myriad of biological processes and their molecular bases, and explore the application of this knowledge to the areas of health, agriculture, industry, and ecology. In this context, more than thirteen bee species have had their genomes sequenced, among which most are from the main tribes of corbiculate Apidae: Apini (*Apis cerana*, *A*. *dorsata*, *A*. *florea*, *A*. *mellifera*), Euglossini (*Eufriesea mexicana*, *Euglossa dilemma*), Bombini (*Bombus terrestris*, *B*. *impatiens*) and Meliponini (*Melipona quadrifasciata*)^3–8^. Meliponini is the more diverse group of bees and comprises over 500 species of generalist pollinators^9^ distributed in the pantropical region^10^. The bees of this group have a non-functional sting and thus are called stingless bees. In the last years, the wild populations of stingless bees are experiencing substantial decline that has been attributed to several factors, including habitat degradation and exposure to toxic substances such as pesticides^11–14^.

The use of gene expression analysis tools has the potential of unravel how specific genes respond to disadvantageous environment conditions and thus allow us to infer the consequences on the organism physiology, development and survival. Thanks to the availability of genomic data, it is now possible to pinpoint the molecular bases of the impact of environmental changes on the stingless bee species through a wide range of techniques. Real-time quantitative PCR (qPCR) is an efficient, simple, and low-cost technique frequently used by molecular biologists to quantify gene expression. The calculation of the relative expression of a target-gene by qPCR is based on the use of reference gene(s) as endogenous control(s). Reference genes should display a constant expression through the experimental conditions, otherwise they would lead to unreliable transcriptional quantification^15^. Historically, the genes used as references for qPCR were those related to basic cell functions that were thought to be expressed at constant rates, the so called “housekeeping genes”. Nonetheless, several studies have shown that biotic and abiotic factors can affect the functioning of the cells or their basic machinery, which would impact the expression of the housekeeping genes. Reliable reference genes can be identified by algorithms such as geNorm^16^, NormFinder^17^, and BestKeeper^18^ that evaluate the stability of genes based on the variance of quantification cycle (Cq) values in each physiological or experimental condition.

Studies on how changes in the environment affect stingless bee populations are required to provide a foundation for the implementation of proper protection measures. Suitable genes for normalization in qPCR were identified in bee species of Apini^19–24^, Bombini^25,26^ and Euglossini^27^ (Supplementary Table S1). Information on stable genes in bees of Meliponini tribe is still missing. Aiming to contribute to molecular studies on *Frieseomelitta varia*, *Melipona quadrifasciata*, and *Scaptotrigona bipunctata* stingless bees, we tested the stability of eight genes previously used as references in insect studies^19–21,23,28–33^ and involved in protein biosynthesis (*elongation factor 1-α*, *ribosomal protein S5*, *ribosomal protein S18*, *ribosomal protein L32*), glucose metabolism (*glyceraldehyde 3-phosphate dehydrogenase*), transcription initiation (*tata-box bind binding protein* and *tata-box binding protein associated factor*) and cytoskeletal structure (*actin*). Here, the expression of these genes was characterized across physiological or experimental conditions related to development, sex, tissues, bacteria injection and pesticide exposure. The choice of the stingless bee species was based on the diversity of their life stories and social organization, on the availability of genomic and transcriptomic sequences for *M*. *quadrifasciata*^4,34^, *F*. *varia*^34,35^ (genome paper submitted) and *S*. *bipunctata* (*in prep*), and on the potential to explore (1) the effects of pesticides on stingless bee populations, (2) the immune response to bacterial challenge, and (3) the diversity of reproductive biology and caste determination mechanisms^36^. We used the RefFinder tool that compiles the results of four methods into a comprehensive ranking based on the stability of the candidate reference genes^37^. This method has been widely used and offer an effective integration of tools for identifying reference genes^38–41^. Our work contributes to studies that aim to investigate the molecular bases of events related to development, sex, tissues, immunity, and pesticide exposure in the three different models of stingless bee species here addressed.

## Results

### Primer evaluation and expression profiles of candidate reference genes

The genomic sequences of the eight candidate reference genes of *F*. *varia*, *M*. *quadrifasciata* and *S*. *bipunctata* were aligned and primers were designed to target similar exonic regions among the three species (Table 1, Supplementary Fig. S1). The specificity of amplification of the eight candidate reference genes was initially tested for each species by conventional PCR and qPCR using a pool of cDNA samples (including all physiological and experimental conditions tested in this study) and genomic DNA. The set of primers were designed to identify possible genomic DNA contamination, as they anneal to different exons and flank intronic regions (Supplementary Fig. S2). The fragments amplified by conventional PCR (amplicons) were visualized by electrophoresis on 2% agarose gels. Amplicons for all genes showed a single band, except for *act* and *tbp* (Supplementary Fig. S3). Specific amplification of the genes *ef1-α*, *gapdh*, *rpl32*, *rps5*, *rps18* and *tbp-af* were subsequently confirmed by a single peak in melting curve analysis (Supplementary Fig. S4-6). In the PCR efficiency calculation, the candidate reference genes displayed high linear regression coefficients that were greater than to 0.99, except *actin* and *tbp* genes for the three species. The PCR amplification efficiency values of *act* and *tbp* were over 110% for all three species, and more than one peak was observed in the melting curve analysis (Supplementary Fig. S7-9**)**. We tried different primer concentrations and annealing temperatures to optimize qPCR, but more than one peak in melting curve analysis was still observed for *act* and *tbp* (data not shown). Besides, *act* seems to have transcript variants in these bee species, making it a gene not suitable for use as a reference gene. Thus, *act* and *tbp* were discarded for further analysis in this work. PCR efficiency values of the other qPCR candidate genes ranged from 90% to 101.3% (Table 2).

**Table 1:** Primer sequences, amplicon sizes and GenBank (NCBI database) accession numbers of candidate reference genes used for qPCR experiments with samples of *F*. *varia*, *M*. *quadrifasciata*, and *S*. *bipunctata*.

Gene	Accession number *F*. *varia*	Accession number *M*. *quadrifasciata*	Accession number *S*. *bipunctata*	Primer sequence (5′-3′)	Amplicon size (bp)
*actin1* (act)*	MN193732	MN687953	MN193747	F: CAAAGCAGGATTTGCAGGAG R: TAAAACGCCCCTTTTGCTTT	135
*elongation factor 1 alpha* *(ef1-α)*	MN193733	MN687951	MN193744	F: GACTGTCGAACGCAAGGAAG R: TCAACACACCGGTTTCAACA	176
*glyceraldehyde 3-phosphate dehydrogenase* *(gapdh)*	MN193734	MN687952	MN193745	F: GTTCAGTGAGCGTGATCCAA R: CTTTGCACCACCTTCCAAAT	124
*ribosomal protein l32* *(rpl32)*	MN193737	MN687948	MN193741	F: CGTAGGCGTTTTAAGGGACA R: ACTCCGTGAGCAATCTCAGC	173
*ribosomal protein s5* *(rps5)*	MN193738	MN687947	MN193740	F: TGTTGACAGGGGACAATCCT R: TGGCCTGATTTACTCGTCGT	147
*ribosomal protein s18* *(rps18)*	MN193739	MN687946	MN193746	F: CGTGCTGGAGAATGTTCTGA R: ATTCGTTCCAAATCCTCACG	179
*tata-box binding protein* *(tbp)*	MN193735	MN687950	MN193743	F: CCCTCTTTTGCAACTCCACA R: GGATCTGCAGAAGCTGGTGT	152
*tata-box binding protein associated factor* *(tbp-af)*	MN193736	MN687949	MN193742	F: TGCTGGACAACCACTTTCTG R: GTGCGGCTAATGAAACCAAT	143

**Table 2:** Amplification efficiencies (E) and correlation coefficients (R^2^) obtained for primers used to amplify reference candidate genes in samples of *F*. *varia*, *M*. *quadrifasciata* and *S*. *bipunctata*. The annealing temperature was 60 °C for all primers and reactions.

Gene	*F. varia*		*M. quadrifasciata*		*S. bipunctata*	
	E (%)	R^2^	E (%)	R^2^	E (%)	R^2^
*act*	115.0	0.700	112.7	0.700	160.4	0.924
*ef1-α*	98.2	0.996	101.0	0.998	91.5	0.997
*gapdh*	97.0	0.997	95.2	0.997	90.2	0.996
*rpl32*	98.9	0.998	95.5	0.993	93.8	0.994
*rps5*	96.1	1.000	100.3	0.996	109.8	0.992
*rps18*	101.3	0.999	96.9	0.995	95.4	0.995
*tbp*	312.3	0.994	218.9	0.880	257.9	0.774
*tbp-af*	99.7	0.992	96.8	0.996	92.9	0.994

**Figure 1:**
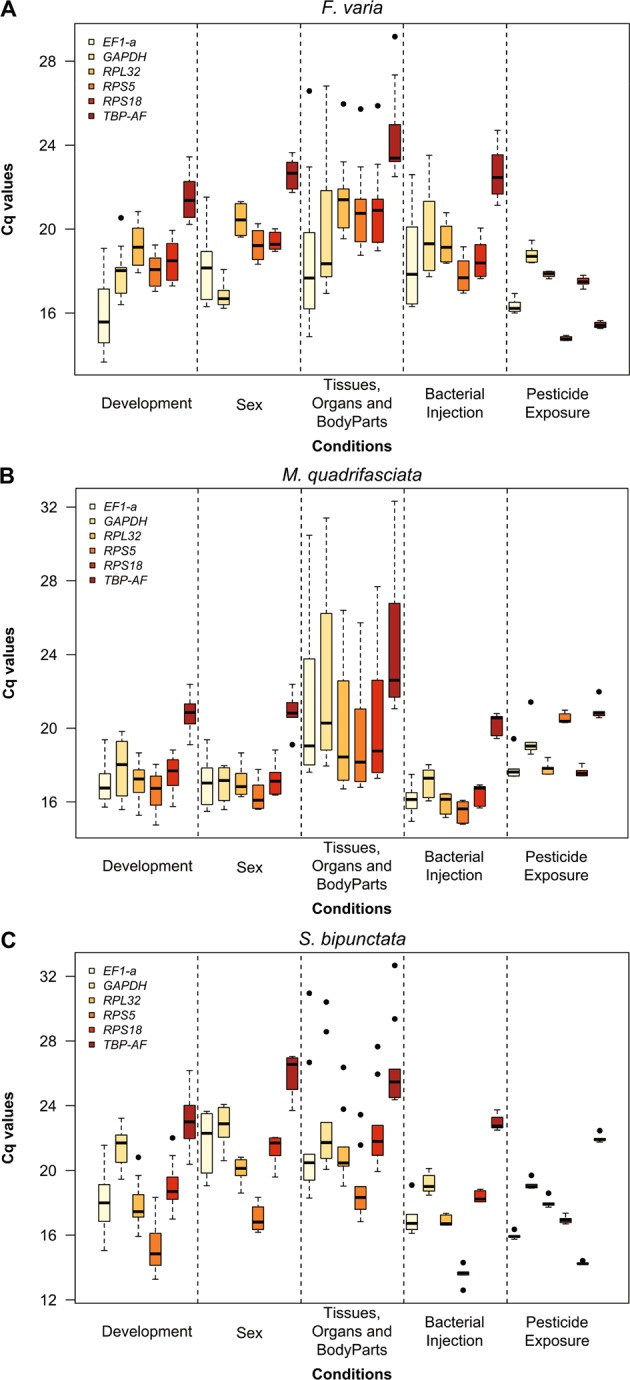
Expression levels (Cq value assessed by qPCR) of candidate reference genes across different experimental contexts using (**A**) *Frieseomelitta varia*, (**B**) *Melipona quadrifasciata* and (**C**) *Scaptotrigona bipunctata*. Box-plots show medians (horizontal lines), 25th to 75th percentiles (boxes), and interquartile ranges (whiskers). The dots indicate the outliers (replicated samples with Cq values above 50% of the interquartile ranges).

Expression levels of the six candidate reference genes were verified in all samples of *F*. *varia*, *M*. *quadrifasciata* and *S*. *bipunctata* to obtain an overview of transcript abundance. A variable Cq value of all the candidates across the physiological and experimental conditions highlighted different expression levels and expression patterns for the three species (Fig. 1, Supplementary Data S1). In *F*. *varia*, Cq values varied from 13.66 (*ef1-α* in different developmental stages) to 29.17 (*tbp-af* among different tissues, organs and body parts). In *M*. *quadrifasciata*, the lowest Cq value was 14.75 (*rps5* in development) and the highest was 32.32 (*tbp-af* in tissues, organs and body parts). In *S*. *bipunctata*, Cq values ranged from 12.60 (*rps5* in bacterial injection) to 32.67 (*tbp-af* in tissues, organs and body parts) (Supplementary Data S1). The *tbp-af* gene showed a higher Cq geo mean in all experimental conditions analyzed for the three species (the Cq geo mean ranged from 15.43 to 26.31), except for pesticide exposure condition of *F*. *varia*, where *gapdh* showed the highest Cq geo mean (18.77) (Supplementary Data S1). The genes with highest Cq variation (standard deviation - SD) in all three species were *ef1-α* and *gapdh*: *ef1-α* showed highest Cq variation during *F*. *varia* and *S*. *bipunctata* development (±Cq SD 1.49 and 1.30, respectively), between sexes of the three bee species (±Cq SD ranged from 1.04 to 1.62), and after bacterial injection into the three bee species (±Cq SD ranged from 0.65 to 2.01); *gapdh* showed highest Cq variation during *M*. *quadrifasciata* development (±Cq SD 1.32), in tissues, organs and body parts of the three bee species (±Cq SD ranged from 2.84 to 4.58) and after pesticide exposure of the three bee species (±Cq SD ranged from 0.20 to 0.69) (Supplementary Data S1). These results show that *ef1-α* and *gapdh* were the least stable genes, but further validation was done using gene expression software tools.

### Stability of candidate reference genes

To determine the stability and rank the candidate reference genes, we used geNorm, NormFinder, Bestkeeper and delta-Ct. These programs are available at the web-tool RefFinder, which also calculated a comprehensive final overall ranking based on the results of these four different algorithms.

geNorm calculates expression stability value (M value) for a candidate reference gene based on the geometric mean of the SD of all studied genes in a pairwise comparison. The reference gene with the lowest M value should be the most stable gene and an M value under 1.5 is suggested by the geNorm software as a criterion for the selection of the reference gene(s)^16^. In our study, M values for all the candidate genes was lower than 1.5 for each physiological and experimental conditions and the three bee species, *F*. *varia* (Table 3), *M*. *quadrifasciata* (Table 4) and *S*. *bipunctata* (Table 5), thus indicating that all genes could act as potential reference genes. Like geNorm, NormFinder calculates stability values based on relative values, and the most stable reference genes are those exhibiting the lowest stability values^17^; however, a cut-off value is not suggested. Bestkeeper calculates the SD value and the coefficient of variation of each candidate gene. An SD greater than 1 indicates high variation of the expression of a gene and, consequently, its instability^18^. Our results demonstrate that some candidate genes are not stable. Gene expression varied most among the tissues, organs and body parts of all the bee species that were analyzed in this study (Tables 3, 4 and 5). The comparative delta-Ct method estimates the most stable reference gene using the SD means by pairwise comparison of two reference genes. An SD below 1 indicates stable gene expression. As observed in our results obtained with Bestkeeper, not all candidate reference genes were stable, and most of the gene expression variations were verified in the tissues, organs and body parts of the bee species (Tables 3, 4 and 5).

**Table 3:** Ranking of six candidate reference genes for *Frieseomelitta varia* based on their expression stability according to analyses by geNorm, NormFinder, BestKeeper, and delta-Ct during development, between sexes, in tissues, and after bacterial injection and pesticide exposure.

Condition	Reference Gene	geNorm		NormFinder		BestKeeper		delta-Ct	
		Stability	Rank	Stability	Rank	Stability	Rank	Stability	Rank
Development	*ef1-α*	0.506	5	0.851	5	1.489	6	0.98	5
	*gapdh*	0.775	6	1.256	6	0.843	2	1.31	6
	*rpl32*	0.132	1	0.066	1	0.892	4	0.54	1
	*rps5*	0.290	4	0.387	4	0.673	1	0.67	4
	*rps18*	0.132	1	0.146	3	0.891	3	0.56	2
	*tbp-af*	0.221	3	0.121	2	0.903	5	0.59	3
Sex	*ef1-α*	0.745	6	1.398	6	1.471	6	1.42	6
	*gapdh*	0.309	4	0.447	4	0.491	2	0.71	4
	*rpl32*	0.120	3	0.065	3	0.548	4	0.53	3
	*rps5*	0.085	1	0.043	1	0.563	5	0.50	1
	*rps18*	0.409	5	0.715	5	0.375	1	0.81	5
	*tbp-af*	0.085	1	0.043	2	0.545	3	0.50	2
Tissues, organs and body parts	*ef1-α*	1.153	6	1.389	6	2.892	5	1.56	6
	*gapdh*	0.948	5	1.347	5	2.947	6	1.53	5
	*rpl32*	0.200	3	0.852	4	1.390	1	1.02	4
	*rps5*	0.078	1	0.556	3	1.552	3	0.89	2
	*rps18*	0.078	1	0.539	2	1.543	2	0.89	1
	*tbp-af*	0.436	4	0.460	1	1.720	4	1.02	3
Bacterial injection	*ef1-α*	0.880	6	1.222	6	2.013	6	1.26	6
	*gapdh*	0.688	5	0.945	5	1.910	5	1.09	5
	*rpl32*	0.084	1	0.542	2	0.843	3	0.72	2
	*rps5*	0.117	3	0.649	4	0.789	1	0.77	4
	*rps18*	0.084	1	0.555	3	0.887	2	0.72	1
	*tbp-af*	0.279	4	0.213	1	1.148	4	0.72	3
Pesticide exposure	*ef1-α*	0.184	5	0.200	5	0.262	5	0.25	5
	*gapdh*	0.217	6	0.260	6	0.334	6	0.28	6
	*rpl32*	0.102	3	0.168	4	0.105	2	0.21	4
	*rps5*	0.065	1	0.141	3	0.078	1	0.19	2
	*rps18*	0.120	4	0.108	2	0.186	4	0.20	3
	*tbp-af*	0.065	1	0.043	1	0.115	3	0.17	1

**Table 4:** Ranking of six candidate reference genes for *Melipona quadrifasciata* based on their expression stability according to analyses by geNorm, NormFinder, BestKeeper, and delta-Ct during development, between sexes, in tissues, and after bacterial injection and pesticide exposure.

Condition	Reference Gene	geNorm		NormFinder		BestKeeper		delta-Ct	
		Stability	Rank	Stability	Rank	Stability	Rank	Stability	Stability
Development	*ef1-α*	1.043	6	1.74	6	0.861	5	1.80	6
	*gapdh*	0.665	5	1.21	5	1.321	6	1.34	5
	*rpl32*	0.257	1	0.13	1	0.774	3	0.75	2
	*rps5*	0.257	1	0.13	2	0.838	4	0.77	3
	*rps18*	0.263	3	0.13	3	0.765	2	0.73	1
	*tbp-af*	0.382	4	0.35	4	0.677	1	0.87	4
Sex	*ef1-α*	0.988	6	1.66	6	1.041	6	1.70	6
	*gapdh*	0.629	5	0.97	5	0.759	5	1.14	5
	*rpl32*	0.145	1	0.07	2	0.696	3	0.70	1
	*rps5*	0.200	3	0.08	3	0.655	1	0.74	3
	*rps18*	0.145	1	0.07	1	0.683	2	0.70	2
	*tbp-af*	0.444	4	0.70	4	0.701	4	0.94	4
Tissues, organs and body parts	*ef1-α*	0.994	5	1.05	4	4.250	5	1.29	4
	*gapdh*	1.164	6	1.39	6	4.580	6	1.51	6
	*rpl32*	0.426	1	0.55	2	3.253	3	0.99	3
	*rps5*	0.698	4	1.21	5	2.744	1	1.32	5
	*rps18*	0.426	1	0.56	3	3.239	2	0.98	2
	*tbp-af*	0.583	3	0.33	1	3.632	4	0.89	1
Bacterial injection	*ef1-α*	0.292	6	0.46	6	0.650	6	0.48	6
	*gapdh*	0.197	5	0.19	5	0.638	5	0.31	5
	*rpl32*	0.094	3	0.18	4	0.465	2	0.26	4
	*rps5*	0.131	4	0.13	3	0.453	1	0.25	3
	*rps18*	0.042	1	0.13	2	0.485	3	0.22	1
	*tbp-af*	0.042	1	0.12	1	0.497	4	0.23	2
Pesticide exposure	*ef1-α*	0.306	5	0.28	3	0.516	5	0.43	5
	*gapdh*	0.416	6	0.62	6	0.686	6	0.64	6
	*rpl32*	0.117	1	0.22	2	0.214	2	0.34	2
	*rps5*	0.140	3	0.32	5	0.237	3	0.39	4
	*rps18*	0.117	1	0.30	4	0.186	1	0.37	3
	*tbp-af*	0.203	4	0.11	1	0.338	4	0.32	1

**Table 5:** Ranking of six candidate reference genes for *Scaptotrigona bipunctata* based on their expression stability according to analyses by geNorm, NormFinder, BestKeeper, and delta-Ct during development, between sexes, in tissues, and after bacterial injection and pesticide exposure.

Condition	Reference Gene	geNorm		NormFinder		BestKeeper		delta-Ct	
		Stability	Rank	Stability	Rank	Stability	Rank	Stability	Rank
Development	*ef1-α*	0.485	5	0.718	5	1.295	6	0.84	5
	*gapdh*	0.799	6	1.399	6	0.970	1	1.43	6
	*rpl32*	0.182	1	0.091	1	1.036	2	0.57	2
	*rps5*	0.266	3	0.221	3	1.152	4	0.67	3
	*rps18*	0.182	1	0.091	2	1.067	3	0.57	1
	*tbp-af*	0.401	4	0.527	4	1.243	5	0.72	4
Sex	*ef1-α*	0.991	6	1.251	6	1.624	6	1.33	6
	*gapdh*	0.643	4	0.643	4	1.002	4	0.97	4
	*rpl32*	0.228	1	0.343	3	0.575	1	0.80	2
	*rps5*	0.819	5	1.212	5	0.715	3	1.28	5
	*rps18*	0.228	1	0.114	1	0.713	2	0.73	1
	*tbp-af*	0.534	3	0.267	2	1.076	5	0.83	3
Tissues, organs and body parts	*ef1-α*	1.223	6	1.561	6	3.106	6	1.69	6
	*gapdh*	0.989	5	1.189	5	2.842	5	1.46	5
	*rpl32*	0.341	1	0.752	3	1.630	2	1.04	3
	*rps5*	0.341	1	0.981	4	1.586	1	1.15	4
	*rps18*	0.414	3	0.537	2	1.889	3	1.00	2
	*tbp-af*	0.585	4	0.341	1	2.039	4	0.99	1
Bacterial injection	*ef1-α*	0.608	6	0.897	6	0.763	6	0.92	6
	*gapdh*	0.243	4	0.151	4	0.487	5	0.51	4
	*rpl32*	0.055	1	0.027	2	0.279	1	0.43	2
	*rps5*	0.450	5	0.884	5	0.343	3	0.91	5
	*rps18*	0.055	1	0.027	1	0.286	2	0.42	1
	*tbp-af*	0.166	3	0.107	3	0.380	4	0.46	3
Pesticide exposure	*ef1-α*	0.202	5	0.217	5	0.131	2	0.26	5
	*gapdh*	0.229	6	0.251	6	0.202	6	0.28	6
	*rpl32*	0.096	3	0.197	4	0.197	5	0.23	4
	*rps5*	0.094	1	0.062	1	0.170	4	0.18	1
	*rps18*	0.141	4	0.112	2	0.054	1	0.21	3
	*tbp-af*	0.094	1	0.141	3	0.163	3	0.20	2

The comparison of the reference gene expression ranking of the four methods revealed variations in each physiological and experimental condition and bee species (Tables 3, 4 and 5). Even so, *rpl32*, *rps5*, *rps18* and *tbp-af* were clearly among the top three stable reference genes in most analyses. It should be noted that Bestkeeper highlighted *gapdh* as a stable gene when we used samples of *F*. *varia* and *S*. *bipunctata* in the development condition, and also when we used *F*. *varia* males and females (sex condition) (Tables 3 and 5). The gene *ef1-α* was considered stable in *M*. *quadrifasciata* and *S*. *bipunctata* treated with pesticides by NormFinder (Table 4) and Bestkeeper (Table 5).

### Comprehensive gene expression ranking and recommended reference genes

As the four programs (geNorm, NormFinder, BestKeeper, delta-Ct) showed different ranking orders of candidate reference genes, we used RefFinder to integrate the rankings and obtain a consensus result in order to recommend the best reference gene(s) for each physiological and experimental condition, and bee species. Based on the comprehensive ranking, *rpls32* and *rps18* were the most stable reference genes in the various developmental stages of *F*. *varia*, *M*. *quadrifasciata* and *S*. *bipunctata* (Table 6). In females and males (sex condition), the genes *rpls32* and *rps18* were highly stable in *M*. *quadrifasciata* and *S*. *bipunctata*, and the genes *rps5* and *tbp-af* were the most stable in *F*. *varia*. In the different tissues, organs and body parts of the three bee species, *rps18*, *rps5* and *tbp-af* were the most stable reference genes (*rps18* and *rps5* in *F*. *varia*, *rps18* and *tbp-af* in *M*. *quadrifasciata*, *rps5* and *tbp-af* in *S*. *bipunctata*). After bacterial injection, *rps18* and *rpl32* were highly stable in *F*. *varia* and *S*. *bipunctata* and *rps18* and *tbp-af* were highly stable in *M*. *quadrifasciata*. After pesticide exposure, the genes *tbp-af* and *rps5* were highly stable in *F*. *varia* and *S*. *bipunctata* and *rpl32* and *rps18* were highly stable in *M*. *quadrifasciata*.

**Table 6:** Comprehensive ranking and best recommended pair of reference genes for accurate normalization of qPCR assays using samples of Meliponini bees under different experimental contexts. The comprehensive ranking is based on gene expression stability of each gene calculated by RefFinder using geNorm, NormFinder, BestKeeper and delta-Ct algorithms.

Condition	Reference Gene	*F. varia*			*M. quadrifasciata*			*S. bipunctata*		
		Comprehensive Ranking		Most stable	Comprehensive Ranking		Most stable	Comprehensive Ranking		Most stable
		Stability	Rank		Stability	Rank		Stability	Rank	
Development	*ef1-α*	5.23	6	rpl32, rps18	5.73	6	rpl32, rps18	5.23	6	rpl32, rps18
	*gapdh*	4.56	5		5.23	5		3.83	4	
	*rpl32*	1.41	1		1.57	1		1.41	1	
	*rps5*	2.83	3		2.21	3		3.22	3	
	*rps18*	2.06	2		2.06	2		1.57	2	
	*tbp-af*	3.08	4		2.83	4		4.23	5	
Sex	*ef1-α*	6.00	6	*rps5, tbp-af*	6.00	6	*rpl32, rps18*	6.00	6	*rps18, rpl32*
	*gapdh*	3.36	5		5.00	5		4.00	4	
	*rpl32*	3.22	3		1.32	1		1.57	2	
	*rps5*	1.50	1		2.28	3		4.40	5	
	*rps18*	3.34	4		1.68	2		1.19	1	
	*tbp-af*	1.86	2		4.00	4		3.08	3	
Tissues, organs and body parts	*ef1-α*	5.73	6	*rps18, rps5*	4.47	5	*rps18, tbp-af*	6.00	6	*rps5, tbp-af*
	*gapdh*	5.23	5		6.00	6		5.00	5	
	*rpl32*	2.63	3		2.06	3		2.06	3	
	*rps5*	2.06	2		3.16	4		2.00	1	
	*rps18*	1.41	1		1.86	1		2.45	4	
	*tbp-af*	2.63	4		1.86	2		2.00	2	
Bacterial injection	*ef1-α*	6.00	6	*rps18, rpl32*	6.00	6	*rps18, tbp-af*	6.00	6	*rps18, rpl32*
	*gapdh*	5.00	5		5.00	5		4.23	4	
	*rpl32*	1.86	2		3.13	4		1.41	2	
	*rps5*	2.63	3		2.45	3		4.40	5	
	*rps18*	1.57	1		1.57	1		1.19	1	
	*tbp-af*	2.63	4		1.68	2		3.22	3	
Pesticide exposure	*ef1-α*	5.00	5	*tbp-af, rps5*	4.40	5	*rpl32, rps18*	3.98	5	*rps5, tbp-af*
	*gapdh*	6.00	6		6.00	6		6.00	6	
	*rpl32*	3.13	3		1.68	1		3.94	4	
	*rps5*	1.57	2		3.66	4		1.41	1	
	*rps18*	3.13	4		1.86	2		2.21	3	
	*tbp-af*	1.32	1		2.00	3		2.06	2	

It is important to highlight that RefFinder tool does not require PCR efficiency values to calculate gene stability and some concern was raised that the outputs of this tool would be thus biased when evaluating the best reference gene(s)^42^. We used geNorm, the most affected algorithm by different PCR efficiencies^42^, to compare the results from the geNorm^PLUS^ (qbasePLUS, version 3.0^43^) and the geNorm from RefFinder. For all conditions and species, both geNorm^PLUS^ and geNorm considered the same three genes as the most stable with slight differences in the position of the genes in the ranking (Supplementary Table S2-4) (an exception was *tbp-af* in the sexes of *S*. *bipunctata*). Thus, for the set of primers and conditions we tested, PCR efficiency is unlikely to be relevant for the identification of the most stable genes.

### Influence of reference gene choice on the relative expression of a target mRNA

**Figure 2:**
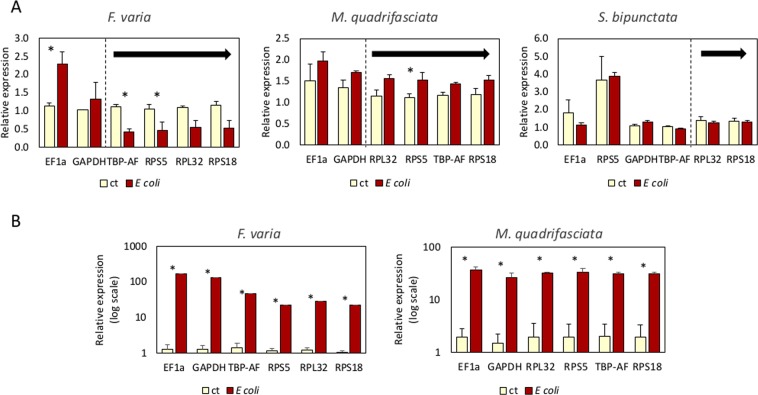
Validation of selected genes in *Frieseomelitta varia*, *Melipona quadrifasciata* and *Scaptotrigona bipunctata*. (**A**) Relative expression of *imd* was analyzed using the six selected reference genes for qPCR normalization in bees injected with *Escherichia coli* versus non-injected ones (control, ct). The first and the second most stable reference genes established for at least one of the algorithms (geNorm, NormFinder, BestKeeper, and delta-Ct) are indicated by arrows. Asterisks indicate p < 0.05 (t-test). Bars represent the means and standard errors of three biological replicates. (**B**) Relative expression (log scale) of *abaecin* was analyzed using the six selected reference genes for qPCR normalization in bees infected with *Escherichia coli* (*E. coli*) versus non-infected ones (control, ct). Asterisks indicate p < 0.05 (t-test). Bars represent the means and standard errors of three biological replicates.

The four most stable and two least stable reference genes were used to normalize the expression of the *immune deficiency* (*imd*) and *abaecin* target genes in the bees submitted to bacterial infection. The extent of variation in *imd* and *abaecin* expression levels was then determined for each reference gene (Fig. 2). In *F*. *varia*, the relative expression of *imd* using the most stable genes was down-regulated after *E*. *coli* injection (*tbp-af*: most stable in NormFinder and *rps5*: most stable in BestKeeper, *t-test*, p < 0.05). However, when using an unstable gene, *ef1-α*, the result was the opposite: expression of *imd* was up-regulated after injection (t-test, p < 0.05). The use of the most stable reference genes in *M*. *quadrifasciata* resulted in up-regulation of *imd* expression (*rps5*: most stable in BestKeeper, *t-test*, p < 0.05). In *S*. *bipunctata*, *imd* relative expression did not differ between control and treatment when normalized with *rpl32* and *rps18*, which were characterized as the most stable genes by each algorithm or in the comprehensive ranking. In general, expression of *imd* did not change substantially after bacterial injection, but the transcriptional levels of the antimicrobial gene *abaecin* was highly up-regulated compared to the non-injected controls. Normalization using all selected candidate reference genes showed the same profile (Fig. 2b). These results indicate the importance of choosing stable reference genes for normalization of target genes with slight expression changes after treatments.

## Discussion

The identification of gold-standard reference genes is crucial to produce reliable qPCR results. The expression of reference genes is used to correct the fluctuations in the target gene expression levels caused by technical variations in the quantity of total RNA or in the cDNA synthesis. Since there is not a universal set of reference genes that are stably expressed in all organisms and under different physiological and experimental conditions, the stability of candidate genes must be tested. Here, we evaluated the stability of six reference genes (*ef1-α*, *gapdh*, *rpl32*, *rps5*, *rps18* and *tbp-af*) in three stingless bee species during their development, in males and females, in tissues, organs and body parts, and after bacterial injection and pesticide exposure.

Our study showed the ribosomal protein genes *rpl32*, *rps5* and *rps18* as highly stable in each of the physiological and experimental conditions for the three stingless bee species and at least one of these genes was among the most stable genes for each condition tested. The genes *rpl32* and *rps18* were most frequent among the genes displaying high stability and were the most stable during development (*F*. *varia*, *M*. *quadrifasciata* and *S*. *bipunctata*), between sexes (*M*. *quadrifasciata* and *S*. *bipunctata*), and after bacterial injection (*F*. *varia* and *S*. *bipunctata*) and pesticide exposure (*M*. *quadrifasciata*) (Table 6). Ribosomal proteins are involved in translation and their genes have been found to be suitable reference genes for studies using bee species and other insects under several experimental conditions.

The *rpl32* gene is highly stable during development^19^ and in nurses and foragers of *A*. *mellifera* (Hymenoptera)^21^, and in different tissues of *Plutella xylostella* (Lepidoptera)^44^. *rps18* is among the most stable genes during the development of *Tribolium castaneum* (Coleoptera)^45^, *Leptinotarsa decemlineata* (Coleoptera)^46^ and *Lipaphis erysimi* (Hemiptera)^47^, and in nurses and foragers of *A*. *mellifera*^19–24^, and also after bacterial infection in *A*. *mellifera*^20^ and fungal infection in *T*. *castaneum*^48^. In addition to *rpl32* and *rps18*, other ribosomal protein genes in insects are stably expressed during development, between tissues, and under different treatment conditions tested^38,44,45,49,50^.

We found the gene *tbp-af* as stably expressed in *F*. *varia* (between sexes and after pesticide exposure), *M*. *quadrifasciata* (among tissues and after bacterial injection), and *S*. *bipunctata* (among tissues and after pesticide exposure). The *tbp-af* gene codifies an evolutionarily conserved protein that composes the complex required for transcription initiation by RNA polymerase II^51^. *tbp-af* has also been shown to be stably expressed between tissues and after JH-treatment of *A*. *mellifera*^19^, and after exposure of *Bemisia tabaci* (Hemiptera) to the pesticides imidacloprid and buprofezin^52^.

Our results showed that the genes *ef1-α* and *gapdh* were the least stable genes in all tested conditions for *F*. *varia* and *M*. *quadrifasciata*. For *S*. *bipunctata*, the pair of least stable genes varied among different conditions: *ef1-α* and *tbp-af* were the least stable during development; *ef1-α* and *gapdh* were the least stable among tissues and after pesticide exposure; *ef1-α* and *rps5* were the least stable between sexes and after bacterial injection. The protein codified by the gene *ef1-α* promotes chain elongation during polypeptide synthesis at the ribosome and *gapdh* codifies a key enzyme in the glycolytic pathway. *ef1-α* was also found to be the least stable gene in *Bombus terrestris* for target gene expression quantifications in tissues and after virus infection^26^, and also during development of the green peach aphid *Myzus persicae* (Hemiptera)^41^. *gapdh* was unstable after insecticide treatment of *Plutella xylostella* (Lepidoptera)^44^, during the development of *Sesamia inferens* (Lepidoptera)^49^, and in the green peach aphid under biotic and abiotic conditions^41^. In contrast, *ef1-α* was shown to be stably expressed across the tissues of *A*. *mellifera*^19^ and *Bombus lucorum*^25,26^, and during development and between tissues of *P*. *xylostella* (Lepidoptera)^44^ and during development of *Diabrotica virgifera* (Coleoptera)^39^. *gapdh* was also considered very stable in several studies with *A*. *mellifera*^19–24^. The different behavior of housekeeping genes among insects, and even among bee species (Table S1), underscores the need to evaluate them as reference genes for each species and physiological or experimental conditions.

The high variation of Cq values observed among tissues, organs and body parts for most candidate reference genes is due to the higher Cq values obtained for ovary samples in all species (Supplementary Data S2). The reason of these variations may be due to some heterogeneity of the samples. All three species belong to a group with variable reproductive capacities in workers, ranging from workers that always activate their ovaries to sterile workers^53^. Workers of *Melipona* and *Scaptotrigona* can lay reproductive haploid eggs, which will develop into males, and throphic eggs, which are larger eggs lacking the nucleus and that serve as food source to the queen^53,54^. *Frieseomelitta* workers are known to lack functional ovaries and be permanently sterile, however, a recent study on the morphology of adult worker ovaries revealed the presence of normal ovarioles, suggesting that these workers may retain some capacity to activate their ovaries^53^. The variety in worker reproduction capacity in these species, as well the variety of ovary activation/degradation grade among the workers of a given species, certainly reflects in the heterogeneity of tissues in the ovaries. Heterogeneous set of samples imposes extra challenges in the search for stable reference genes^55^. Yet, our results of geNorm^PLUS^ revealed average gene stability values (M) ≤ 0.5 (Supplementary Fig. 10), indicating that the reference genes are stable for this set of biological samples (fat body, head and ovaries). Other candidate reference genes should be evaluated only if average M-values are >1, that indicate low reference gene stability^55^.

All candidate reference genes were tested using RT-qPCR assay to evaluate their influence on the *imd* gene expression in *F*. *varia*, *M*. *quadrifasciata* and *S*. *bipunctata* infected with bacteria. *imd* is a member of the Imd pathway, one of the main pathways of immune response in insects^56^. The suitability of the reference genes for calculating the target gene (*imd*) expression levels was more evident in *F*. *varia* infected with bacteria. In this case, the use of highly stable reference genes (*tbp-af* and *rps5*) evidenced down-regulation of *imd* expression in infected bees whereas the use of one of the least stable genes (*ef1-α*) pointed to up-regulation of *imd* expression after infection (Fig. 2). Down-regulation of *imd* expression was also observed in *A*. *mellifera* infected with *E*. *coli*^57^. Our experiments also highlighted that in *M*. *quadrifasciata* and *S*. *bipunctata*, *imd* is mildly affected by bacterial injection, thus suggesting a distinct immune response in comparison to *F*. *varia*. Different responses to bacterial infection in the recognition and signaling genes from the immune pathways have also been observed between *A*. *mellifera* and *Bombus terrestris*, but the up-regulation of antimicrobial peptides such as *abaecin*, *hymenoptaecin* and *defensin* is always observed is these bees^57–60^. Yet, up-regulation of *abaecin* was detected in injected *F*. *varia* and *M*. *quadrifasciata* regardless of the candidate reference gene used to normalize *abaecin* expression levels. This result agrees with the observations that pronounced differences in expression levels of target gene are more easily detected and less prone to be affected by the choice of reference genes^55^.

This is the first study to validate reference genes for qPCR analysis in stingless bees and provides new resources for research on this diverse and important pollinator group. Recently, more than ten bee genomes have become available^3,4,6^ and allow the exploration of comparative genomic approaches to better understand the multifaceted aspects of bee biology and evolution. Yet, few studies evaluating candidate reference genes in bees were done (Table S1). Here, we used robust tools to evaluate the stability of the candidate reference genes in a comprehensive set of physiological and experimental conditions. Thus, our results have the potential to contribute to a wide range of molecular studies. In general, the ribosomal protein genes *rpl32*, *rps18* and *rps5*, and *tbp-af* showed the highest stability in the stingless bees.

## Material and Methods

### Sample collection

#### Stingless bees

*F*. *varia*, *M*. *quadrifasciata* and *S*. *bipunctata* specimens were sampled directly from colonies located at the University of São Paulo, in Ribeirão Preto and in São Paulo, Brazil. We tested the candidate reference genes using different developmental stages, tissue (fat body), organ (ovaries) and body part (head), both sexes, and different experimental conditions, i.e., immune stimulation by bacterial injection and pesticide exposure. Details of sample collection are described below (see also Supplementary Data S2). For each species and condition, samples were stored in TRIzol ® Reagent (Invitrogen) and kept at −80 °C until RNA extraction. Whole individuals were processed for RNA extraction, except when tissues, organs and body parts were collected.

#### Developmental stages

*F*. *varia*, *M*. *quadrifasciata* and *S*. *bipunctata* workers were sampled throughout development including last instar larvae (defecating larvae, DL), pupae (white-eyed pupae, Pw), newly-emerged (NE) and foragers (FOR). DL have empty intestines^61^. Newly-emerged bees (<24 h-old) were obtained from pieces of brood combs taken from the nest and kept in an incubator (28 °C) for few days. Foragers were collected in the entrance of the nest carrying pollen in the hindlegs. For each species and developmental stage, we sampled three individuals for further analysis.

#### Tissues, organs and body parts

Fat body, head and ovaries from *F*. *varia* FOR, *M*. *quadrifasciata* NE and *S*. *bipunctata* NE were dissected in RNase-free 0.9% NaCl solution. Each sample of fat body or head was a pool obtained from three to six individuals of *F*. *varia* or *S*. *bipunctata*. Eighteen to 22 pairs of ovaries of *F*. *varia* and 11 to 13 pairs of *S*. *bipunctata* were pooled to compose each ovary sample. For *M*. *quadrifasciata*, only one individual was used to prepare each sample due to the bigger size of this stingless bee. Pooled (*F*. *varia* and *S*. *bipunctata*) or individual (*M*. *quadrifasciata*) samples were collected in triplicates.

#### Female vs Male

NE females of the three stingless bee species were compared with males. NE females and *M*. *quadrifasciata* males (<24 h-old) were collected as described above for NE females. Age-specific males were obtained by marking NE males on the thorax with a paint marker, returning them to experimental hives, and collecting them after seven days (*F*. *varia*) or after eight days (*S*. *bipunctata*). Three males were collected for each species.

#### Bacterial injection

Ten NE workers of *F*. *varia*, *M*. *quadrifasciata* and *S*. *bipunctata* in a total of 30 bees were separated in groups of five individuals. One group of each species was injected with a bacterial suspension of *Escherichia coli* DH5α whereas the other group did not receive any treatment (control group). *F*. *varia* workers were injected in the thorax with 1 µL of bacterial suspension in 0.9% NaCl (5 × 10^3^ bacteria/µL) using a Nanofil 10 µL syringe (World Precision Instruments, Sarasota, FL, USA) and a needle with a 0.11 mm outer diameter. *M*. *quadrifasciata* workers were injected in the abdomen (between the 5^th^ and 6^th^ abdominal segments) with 1 µL of bacterial suspension (5 × 10^4^ bacteria/µL) using a Hamilton syringe and a needle with a 0.47 mm outer diameter. *S*. *bipunctata* workers were injected in the thorax with 1 µL of bacterial suspension (5 × 10^3^ bacteria/µL) using a Hamilton 701 N 10 µL syringe (Hamilton®, Reno, NV, USA) and a needle with a 0.47 mm outer diameter. The bacterial concentration used for each species was estimated based on the respective body weight. It is known that injection of 5 × 10^4^ bacteria/µL is enough to activate the immune response in honey bees^62^; thus, we used the same concentration to inoculate *M*. *quadrifasciata*, which has a body weight equivalent to *A*. *mellifera*. *F*. *varia* and *S*. *bipunctata* are about 10-fold smaller than *M*. *quadrifasciata* and the bacterial concentration used for injections was 10-fold lower. The syringe and needle types used to administer the injections were chosen according to cuticle hardness (softer in *F*. *varia*). Only injection *per se* is enough to trigger the honey bee immune response^60^. Thus, immune stimulation was provoked by both injection and bacterial infection. Both injected and control groups of the three species were kept in the incubator at 28 °C with food (50% sucrose solution in water) and water *ad libitum*. The bees were individually collected (n = 3 for each injected and control group) six hours after the bacterial injection.

#### Treatment with imidacloprid pesticide

To produce a primary stock solution, 10 mg of imidacloprid (Sigma Aldrich) was added to 500 µL of acetone, with a subsequent dilution in distilled water to a concentration of 1000 ng active ingredient (a.i.)/µL. This aqueous stock solution was further diluted to prepare the solutions for topical applications. NE workers of *F*. *varia* and *S*. *bipunctata* received on the thorax a topical application of 1 µL of 25.2 ng a.i./µL, a dose tested in *Scaptotrigona postica*^63^, whereas NE workers of *M*. *quadrifasciata* received 1 µL of 2.01 ng a.i./µL, a dose also previously tested in these bees^64^. The control bees received topically 1 µL of water on the thorax. All the bees (n = 5 for each treatment) were maintained in Petri dishes under darkness in an incubator at 28 °C with food (50% sucrose solution in water) and water *ad libitum*. After 24 h, the bees were collected. At this time only alive and active bees were collected (n = 3 for each treated and control group).

### RNA extraction and cDNA synthesis

Extraction of total RNA was performed according to the Trizol® manufacturer’s protocol. To increase total RNA precipitation of ovary samples, 1 µg of Molecular Biology Grade Glycogen (Sigma-Aldrich) was added to the isopropanol step and samples were kept at −20 °C for 24 to 48 hours. For tissue samples (fat body, head and ovary) 1 µg of total RNA was used for cDNA synthesis. For all the other samples (DL, Pw, NE, FOR, male, NE workers injected with *E*. *coli* and their non-injected controls, NE workers treated with pesticide and their non-treated controls), we used 3 µg of total RNA. cDNA synthesis was performed using SuperScript™ II (Invitrogen) and oligo(dT)_12–18_, except for the developmental stages (DL, Pw, RE, FOR) and the experiment of bacterial injection in *F*. *varia* for which SuperScript™ III (Invitrogen) and oligo(dT)_20_ were used. In both cases, cDNA synthesis was performed according to manufacturer’s protocol.

### Primer design and validation by conventional PCR

The sequences of the genes *act*, *ef1-α*, *gapdh*, *rpl32*, *rps5*, *rps18*, *tbp* and *tbp-af* were retrieved from the recently sequenced genome of *F*. *varia* (The genome assembly was submitted to NCBI under BioProject PRJNA528016) and from the genome of *M*. *quadrifasciata* deposited in the Hymenoptera Genome Database^4,65^. *S*. *bipunctata* sequences were identified in a transcriptome dataset (not published). The structural organization of open reading frames and putative exon/intron splice sites of the candidate reference genes were inferred by the annotation tool Artemis version 16.0.0^66^ (Supplementary Fig. S2). The sequences of the genes were deposited in the NCBI data bank (accession numbers in Table 1). Intron-spanning primers were designed based on *F*. *varia* gene sequences (Supplementary Fig. S2) using Primer3 software (bioinfo.ut.ee/primer3–0.4.0/). Melting temperatures between 60 °C and 61 °C, and amplicon length ranging from 100 to 190 bp were the restrictive parameters for primer selection. Other parameters were kept at the default setting. A maximum of two mismatches between the primer and the target sequence of *M*. *quadrifasciata* or *S*. *bipunctata* was allowed. We carefully checked the position of the mismatches in the primer sequence to avoid mismatches in the last five nucleotides of the 3′end. The only exception was the *tbp-af* forward primer for *S*. *bipunctata*, which contained a mismatch at the second last position^67^ (Supplementary Fig. S1). All genes, accession numbers, primer sequences and amplicon sizes used in this study are listed in Table 1.

Conventional PCRs were done to check amplification from complementary DNA (cDNA) and genomic DNA (gDNA) templates. Amplifications were carried out using 2x PCR Master Mix (Promega) and 0.6 pmol of each primer, under the following PCR regime: 95 °C for 5 min; 40 cycles of 95 °C for 30 s, 60 °C for 30 s, 72 °C for 45 s; and a final extension step of 72 °C for 7 min. The PCR products were checked by electrophoresis on 2% agarose gels stained with UniSafe Dye 20.000 × (Uniscience), visualized and documented in Kodak 1D Image Analysis program, version 3.6.2 (Eastman Kodak Co., Rochester, NY).

### Real-time quantitative PCR

The real-time quantitative PCR (qPCR) assays were performed using the StepOnePlus™ Real-Time PCR System (Applied Biosystems). Amplifications were carried out in 15 μL reaction solutions containing 7.5 μL 2x qPCRBIO SyGreen Mix Separate-ROX (PCR Biosystems), 1.5 μL first-stranded cDNA (diluted 1:10), 6 ρmol/μL of each specific primer and 4.8 μL water. PCR conditions were 95 °C for 2 min followed by 40 cycles of 95 °C for 5 s and 60 °C for 25 s. The specificity of each pair of primers was checked by melting curve analysis (95 °C for 15 s, 60 °C for 1 min and a continuous raise in temperature to 95 °C at 0.3 °C/s ramp rate followed by 95 °C for 15 s). To check reproducibility, each assay was performed with technical triplicates for each of the three biological samples. PCR efficiency values (E) were calculated for each gene and bee species from the given slope after running standard curves and following the formula E = (10^(−1/slope)^−1)×100.

### Evaluation of reference gene expression stability

To determine the stability of candidate reference genes we used RefFinder^37^. RefFinder is a web-based analysis tool that integrates four algorithms: geNorm^16^, NormFinder^17^, BestKeeper^18^, and delta-Ct^68^. Based on the rankings generated by each algorithm, a weight is assigned to each gene and geometric means of the gene weights are calculated for a comprehensive final ranking. Candidate genes having lower mean weights are considered transcriptionally stable and can be used as ideal reference genes.

### Validation of reference gene selection

To evaluate each candidate reference gene for qPCR assays using *F*. *varia*, *M*. *quadrifasciata* and *S*. *bipunctata* samples, we measured the expression of the *imd* and *abaecin* gene in adult workers treated with *E*. *coli* and their respective untreated controls. *imd* gene encodes a protein that plays a role in the Imd pathway, which controls antibacterial defense^57^. Primer sequences for *imd* (forward 5′AAC AAC CGA TGC AAA ACC TG 3′ and reverse 5′ TCG TTG TTT TCG GTT CAT CA 3′) and for *abaecin* (*F*. *varia* forward 5′-GAA GGT AAC GAC GTT TAT TTT CG-3′, reverse 5′ TGG AAA CGG ATG TCG TTG TA 3′; *M*. *quadrifasciata* forward 5′ ATG CGC GAT ATT TGC GAT A 3′, reverse 5′TTT TCG GAT TGA ATG GTC CT 3′) were designed using Primer3 software, following the same parameters described in the previous section. The relative transcripts levels of *imd* and *abaecin* were calculated for each bee species using the 2^−ΔΔCT^ method^69^ and the six candidate reference genes. Student’s *t-*test was performed to compare treated and control groups with significance reported for p < 0.05.

## Supplementary information

Supplementary Material

Dataset1

Dataset2
