# Supplementary material for: Evaluation of reference genes for gene expression analysis by real-time quantitative PCR (qPCR) in three stingless bee species (Hymenoptera: Apidae: Meliponini)
Source: Sci Rep. 2019 Nov 27;9:17692. doi: 10.1038/s41598-019-53544-0 (PMC6881334; doi:10.1038/s41598-019-53544-0)
Supplement: Supplementary file 1 — Supplementary Material [file 41598_2019_53544_MOESM1_ESM.docx]

**Evaluation of reference genes for gene expression analysis by real-time quantitative PCR (qPCR) in three stingless bee species (Hymenoptera: Apidae: Meliponini)**

**Authors:** Flávia Cristina de Paula Freitas, Thiago da Silva Depintor, Lucas Trevisoli Agostini, Danielle Luna-Lucena, Francis Morais Franco Nunes, Márcia Maria Gentile Bitondi, Zilá Luz Paulino Simões, Anete Pedro Lourenço


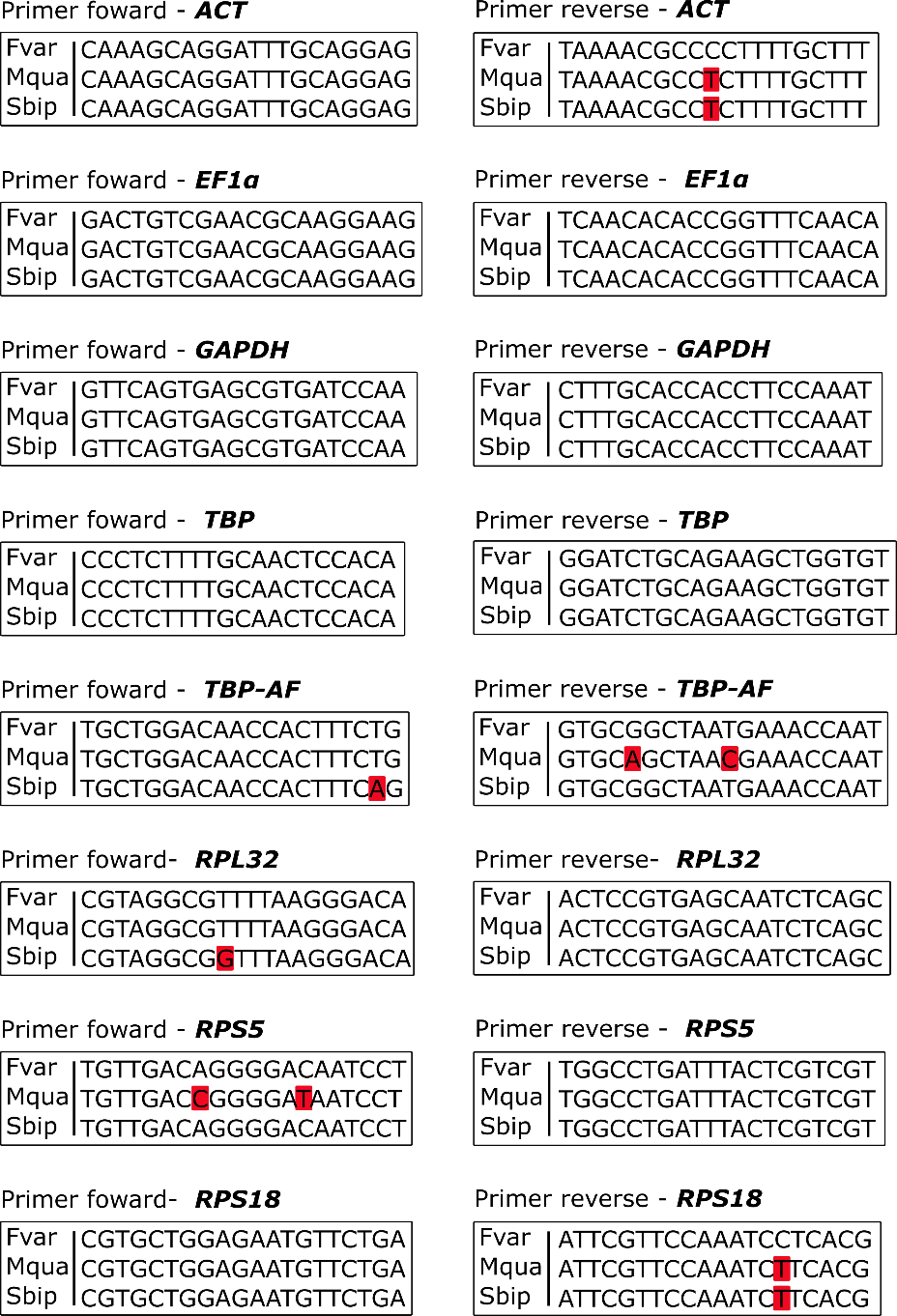


**Figure S1** – Alignments of Forward and Reverse primer sequences (5’ to 3’) designed for each candidate reference gene, highlighting in red nucleotide mismatches found between *Frieseomelitta varia* (Fvar), *Melipona quadrifasciata* (Mqua), and *Scaptotrigona bipunctata* (Sbip). *ACT* (actin), *EF1α* (elongation factor 1-α), *GAPDH* (glyceraldehyde 3-phosphate dehydrogenase), *TBP* (TATA-box binding protein), *TBP-AF* (TATA-box binding protein associated factor), *RPL32* (ribosomal protein L32), *RPS5* (ribosomal protein S5), *RPS18* (ribosomal protein S18).


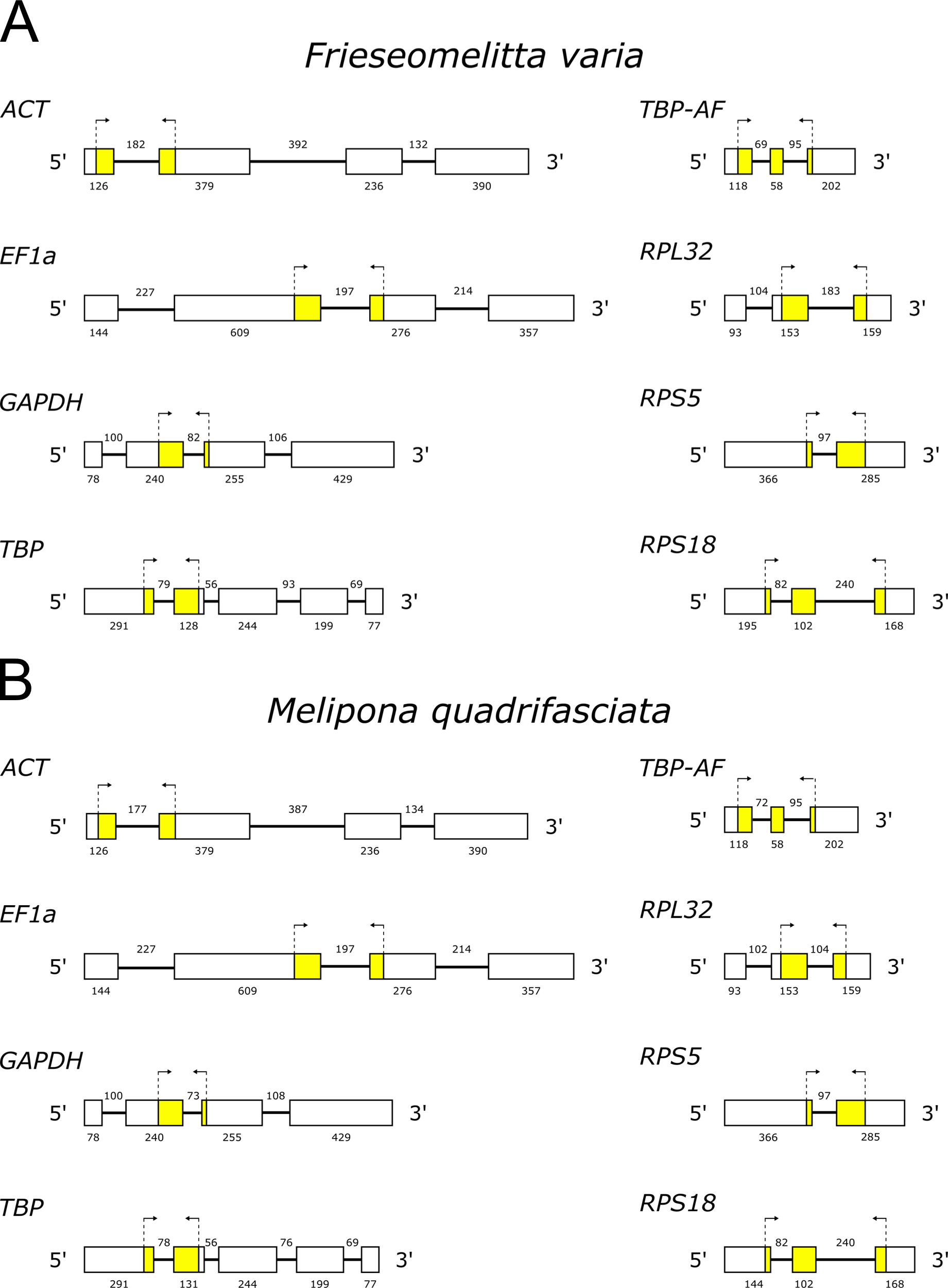


**Figure S2** - Gene architecture of candidate reference genes in (A) *Frieseomelitta varia*, and (B) *Melipona quadrifasciata*. Boxes represent exons, box-connecting lines represent introns, and their respective length in base pairs (bp) are noted accordingly. Primers are indicated by arrows and amplification region from cDNA are highlighted in yellow. *ACT* (actin), *EF1α* (elongation factor 1-α), *GAPDH* (glyceraldehyde 3-phosphate dehydrogenase), *TBP* (TATA-box binding protein), *TBP-AF* (TATA-box binding protein associated factor), *RPL32* (ribosomal protein L32), *RPS5* (ribosomal protein S5), *RPS18* (ribosomal protein S18).


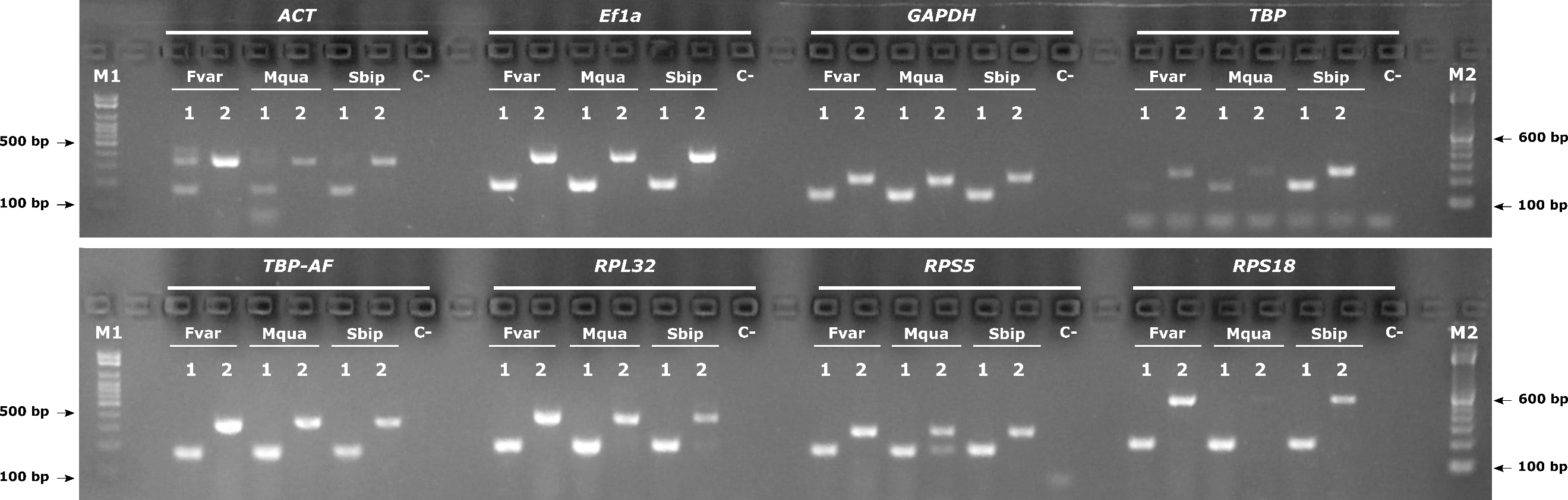


**Figure S3** - PCR amplification specificity of primer pairs designed for eight candidate reference genes: *ACT* (actin), *EF1α* (elongation factor 1-α), *GAPDH* (glyceraldehyde 3-phosphate dehydrogenase), *TBP* (TATA-box binding protein), *TBP-AF* (TATA-box binding protein associated factor), *RPL32* (ribosomal protein L32), *RPS5* (ribosomal protein S5), *RPS18* (ribosomal protein S18). PCR product visualized by electrophoresis in 2% agarose gels stained with UniSafe Dye (20,000x, Uniscience Corp.), visualized and documented in Kodak 1D Image Analysis program, version 3.6.2 (Eastman Kodak Co., Rochester, NY), with 3-5s of exposure. The gel image was not edited and the labels were added to the image in Microsoft Power Point. M1: LowRanger 100 bp DNA Ladder (Norgen Biotek Corp.). M2: TrackIt™ 100 bp DNA ladder (Invitrogen™). 1 - Complementary DNA (cDNA) template. 2 - Genomic DNA (gDNA) template. Fvar - *Frieseomelitta varia*. Mqua - *Melipona quadrifasciata*. Sbip - *Scaptotrigona bipunctata*. C-: Non-template control.


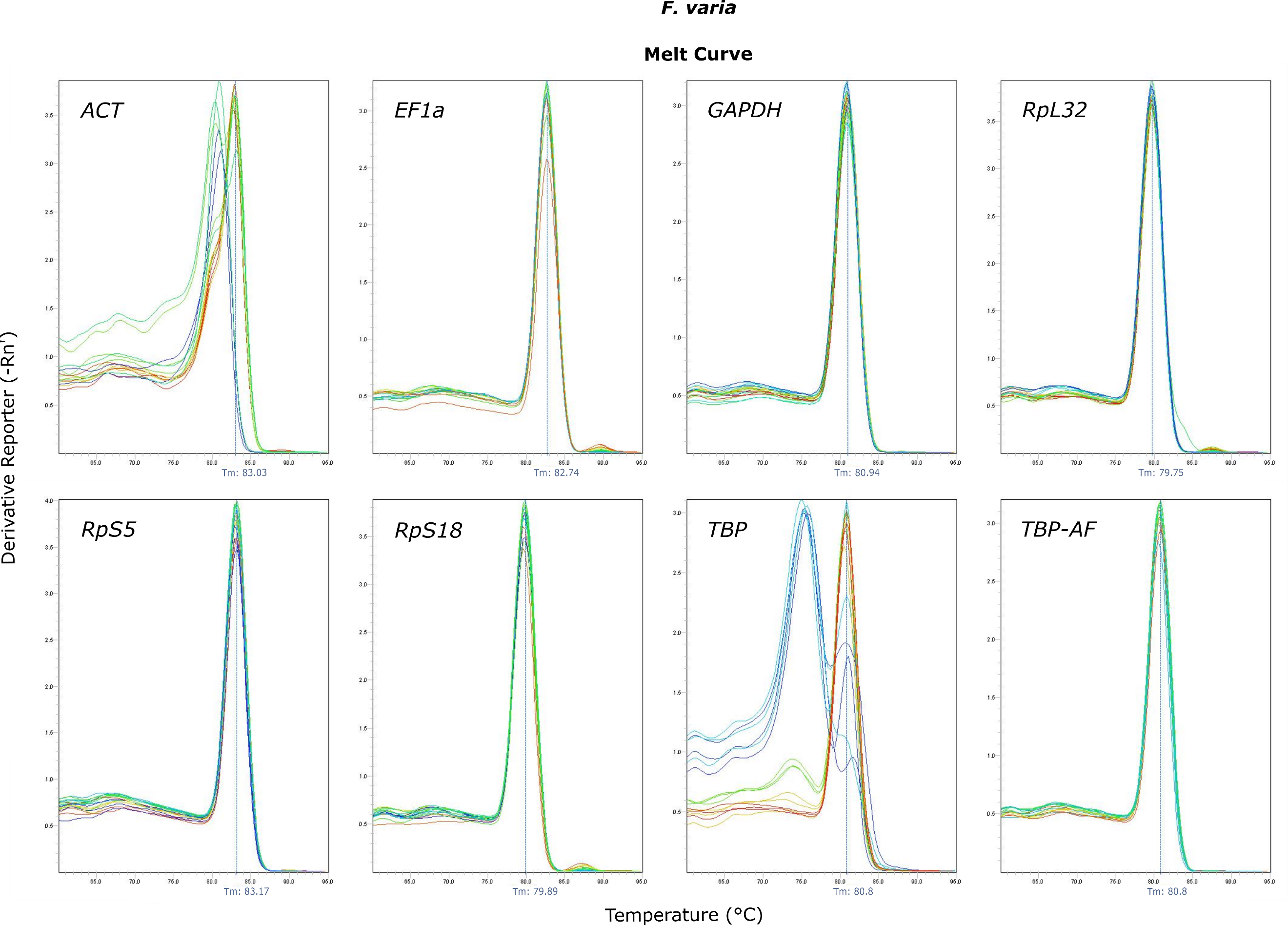


**Figure S4** - Melting curves of eight candidate reference genes indicating specificity, repeatability, and accuracy of each primer pair used for qPCR assays using *Frieseomelitta varia* samples. *ACT* (actin), *EF1α* (elongation factor 1-α), *GAPDH* (glyceraldehyde 3-phosphate dehydrogenase), *TBP* (TATA-box binding protein), *TBP-AF* (TATA-box binding protein associated factor), *RPL32* (ribosomal protein L32), *RPS5* (ribosomal protein S5), *RPS18* (ribosomal protein S18).


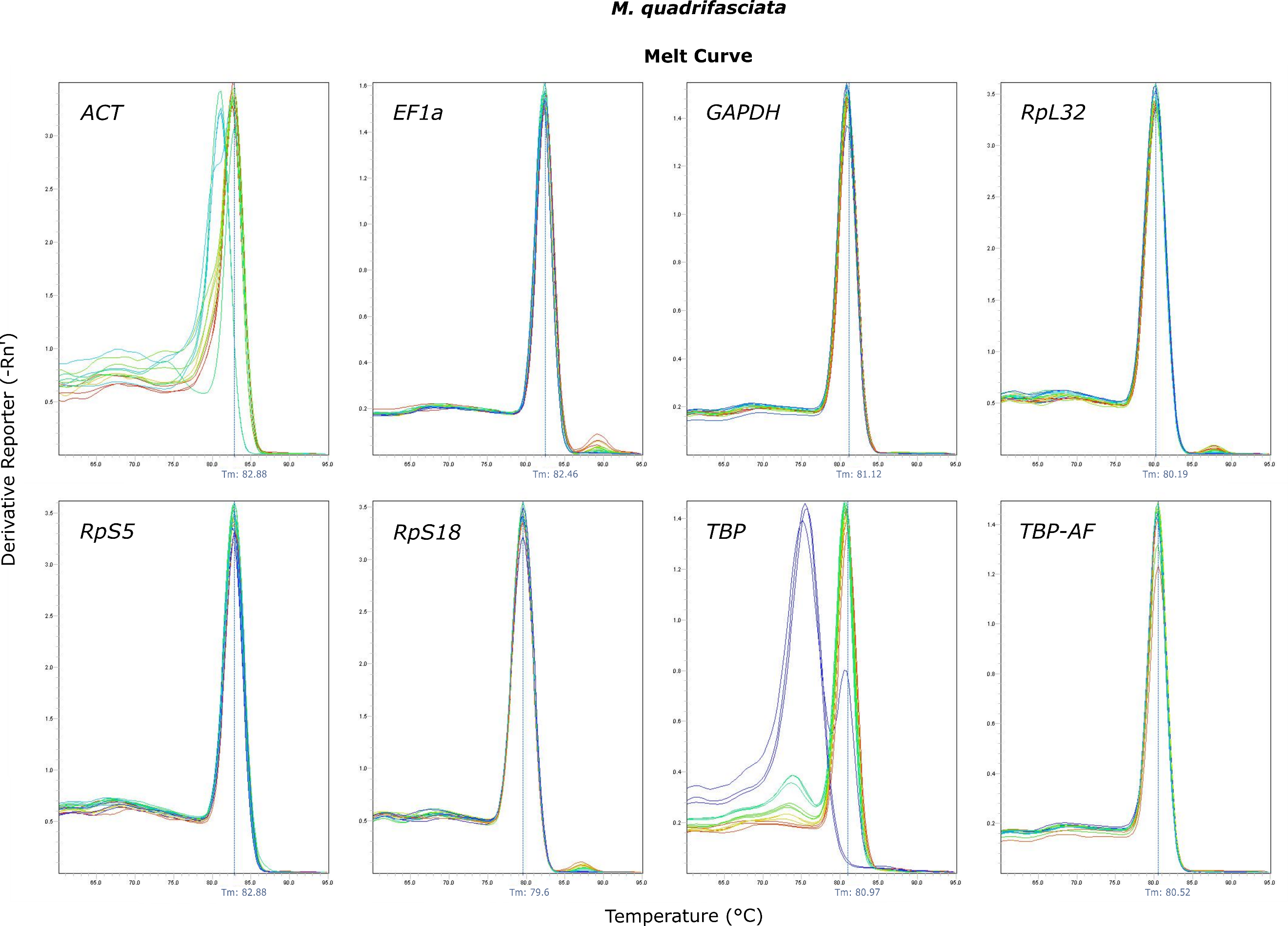


**Figure S5** - Melting curves of eight candidate reference genes indicating specificity, repeatability, and accuracy of each primer pair used for qPCR assays using *Melipona quadrifasciata* samples. *ACT* (actin), *EF1α* (elongation factor 1-α), *GAPDH* (glyceraldehyde 3-phosphate dehydrogenase), *TBP* (TATA-box binding protein), *TBP-AF* (TATA-box binding protein associated factor), *RPL32* (ribosomal protein L32), *RPS5* (ribosomal protein S5), *RPS18* (ribosomal protein S18).


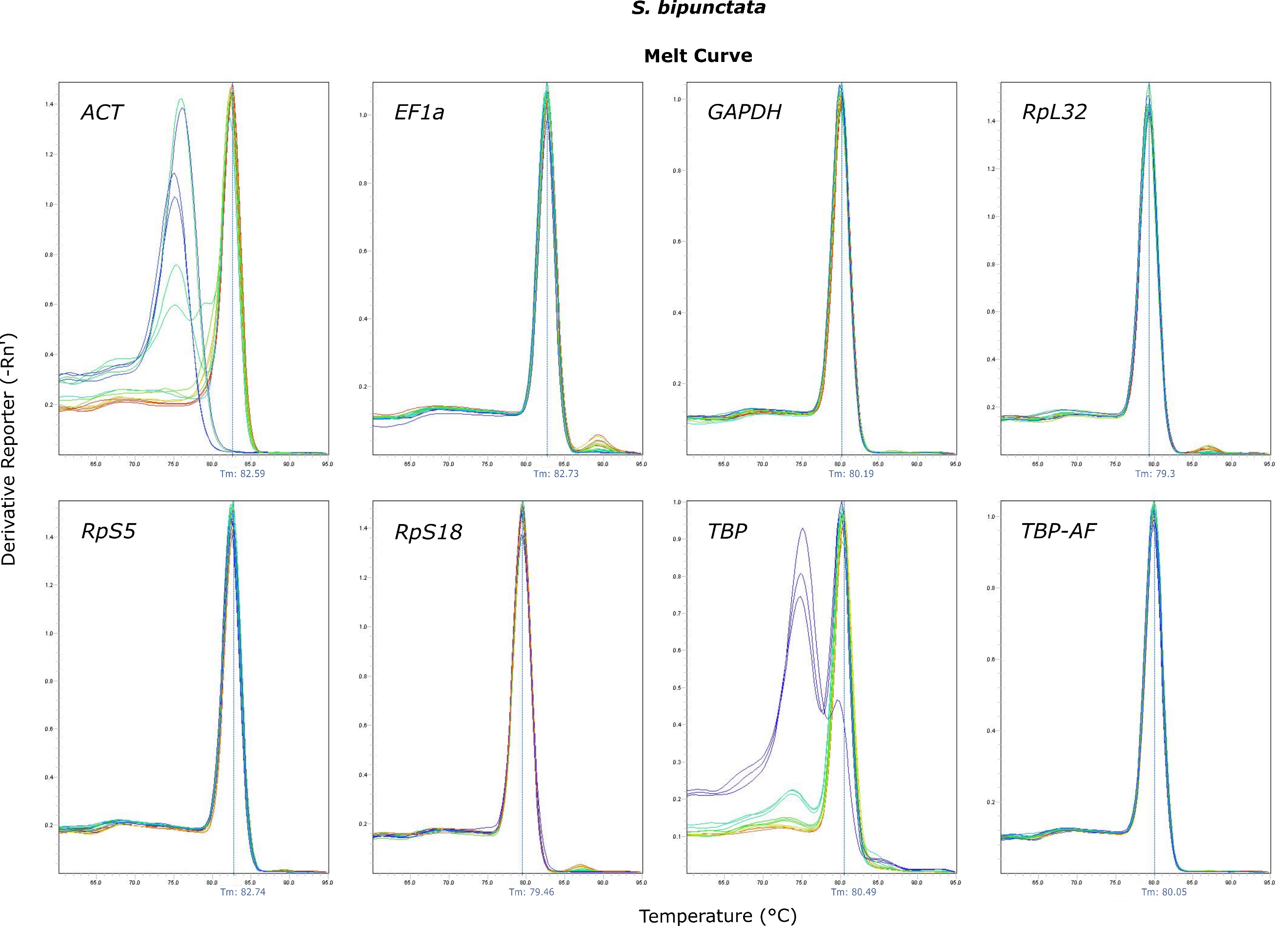


**Figure S6** - Melting curves of eight candidate reference genes indicating specificity, repeatability, and accuracy of each primer pair used for qPCR assays using *Scaptotrigona bipunctata* samples. *ACT* (actin), *EF1α* (elongation factor 1-α), *GAPDH* (glyceraldehyde 3-phosphate dehydrogenase), *TBP* (TATA-box binding protein), *TBP-AF* (TATA-box binding protein associated factor), *RPL32* (ribosomal protein L32), *RPS5* (ribosomal protein S5), *RPS18* (ribosomal protein S18).


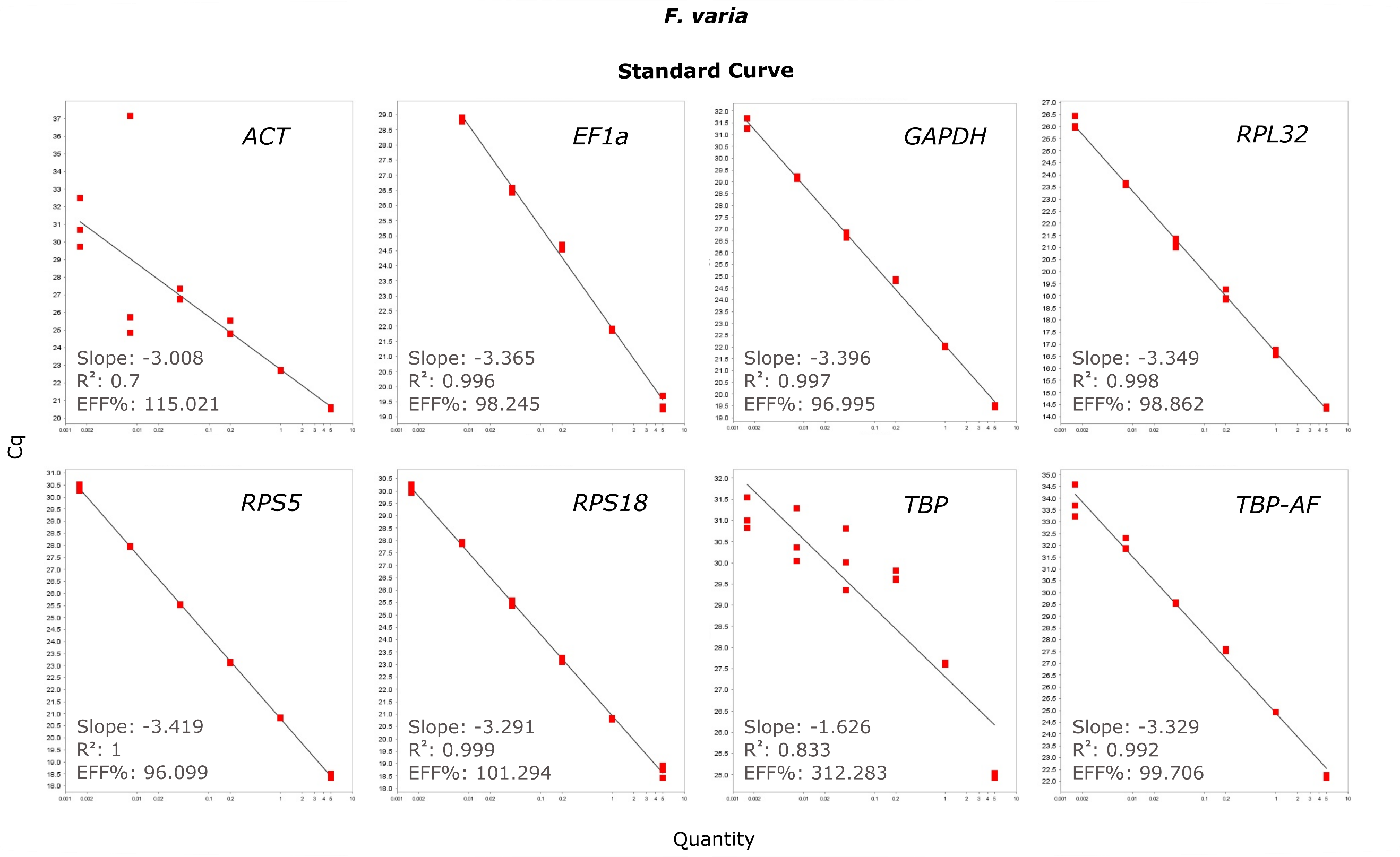


**Figure S7**- Standard curves of eight candidate reference genes indicating slope, R^2^ and percentage of efficiency (EFF%) for each primer pair used for qPCR assays using *Frieseomelitta varia* samples. *ACT* (actin), *EF1α* (elongation factor 1-α), *GAPDH* (glyceraldehyde 3-phosphate dehydrogenase), *TBP* (TATA-box binding protein), *TBP-AF* (TATA-box binding protein associated factor), *RPL32* (ribosomal protein L32), *RPS5* (ribosomal protein S5), *RPS18* (ribosomal protein S18).


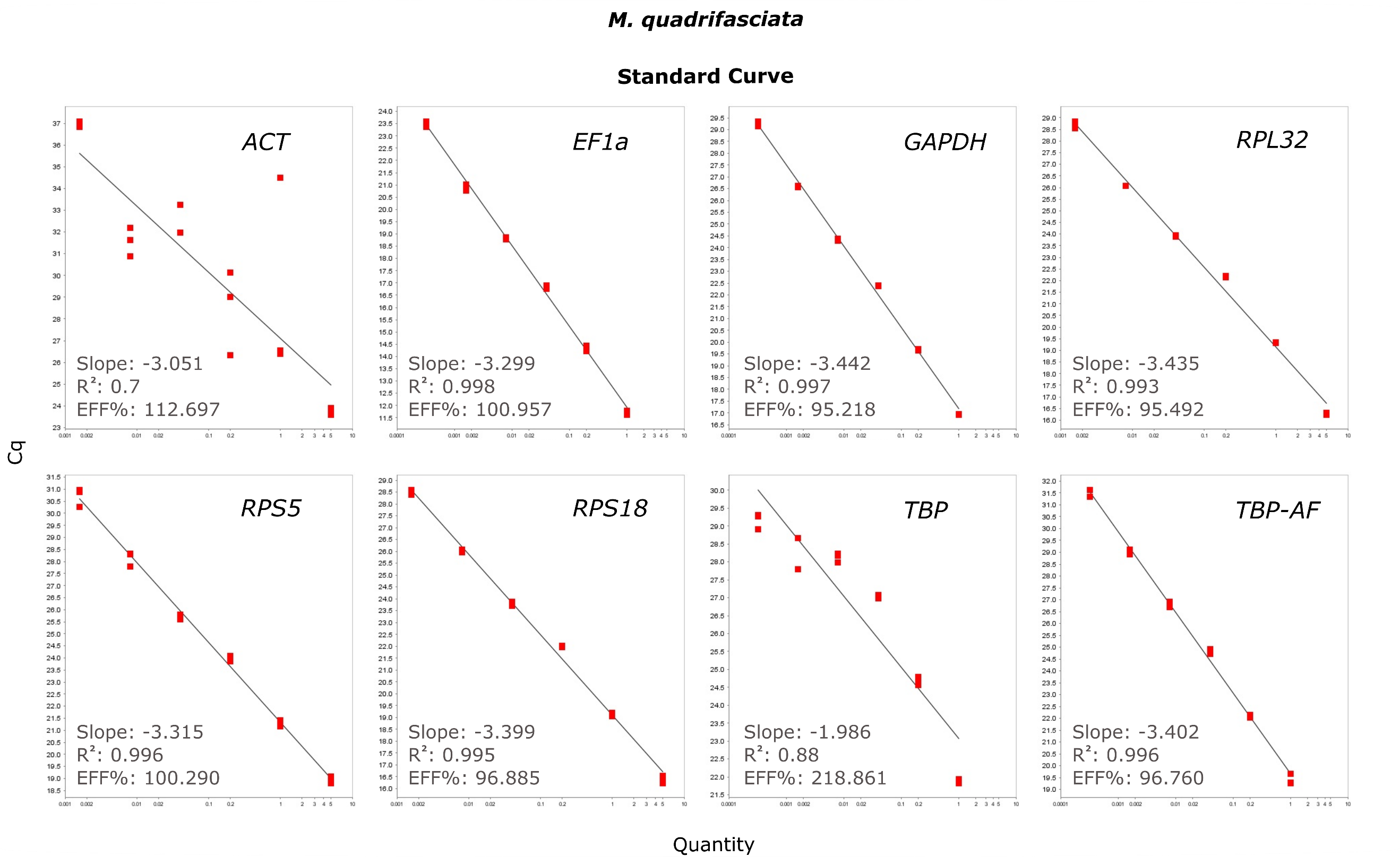


**Figure S8**- Standard curves of eight candidate reference genes indicating slope, R^2^ and percentage of efficiency (EFF%) for each primer pair used for qPCR assays using *Melipona quadrifasciata* samples. *ACT* (actin), *EF1α* (elongation factor 1-α), *GAPDH* (glyceraldehyde 3-phosphate dehydrogenase), *TBP* (TATA-box binding protein), *TBP-AF* (TATA-box binding protein associated factor), *RPL32* (ribosomal protein L32), *RPS5* (ribosomal protein S5), *RPS18* (ribosomal protein S18).


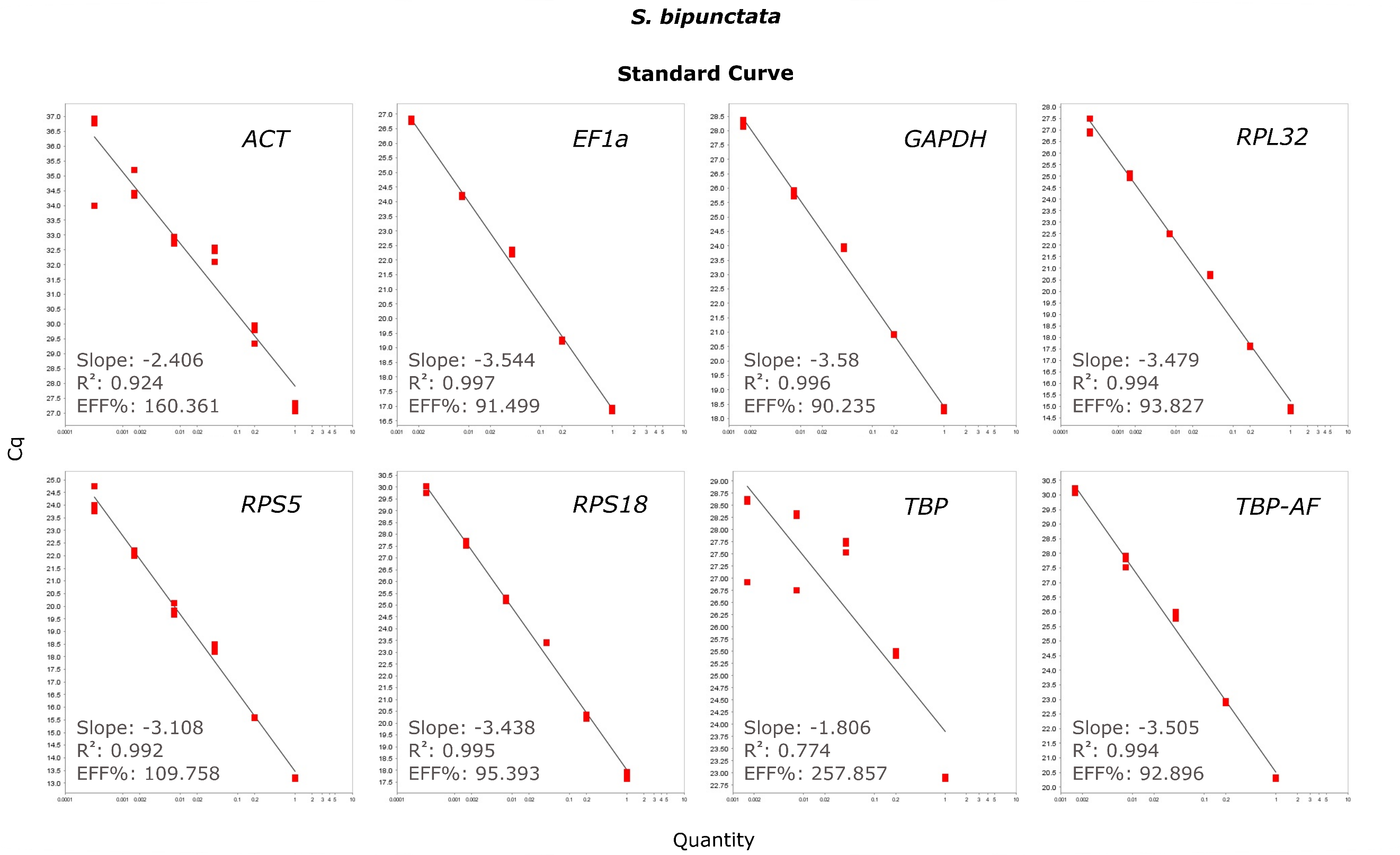


**Figure S9**- Standard curves of eight candidate reference genes indicating slope, R^2^ and percentage of efficiency (EFF%) for each primer pair used for qPCR assays using *Scaptotrigona bipunctata* samples. *ACT* (actin), *EF1α* (elongation factor 1-α), *GAPDH* (glyceraldehyde 3-phosphate dehydrogenase), *TBP* (TATA-box binding protein), *TBP-AF* (TATA-box binding protein associated factor), *RPL32* (ribosomal protein L32), *RPS5* (ribosomal protein S5), *RPS18* (ribosomal protein S18).


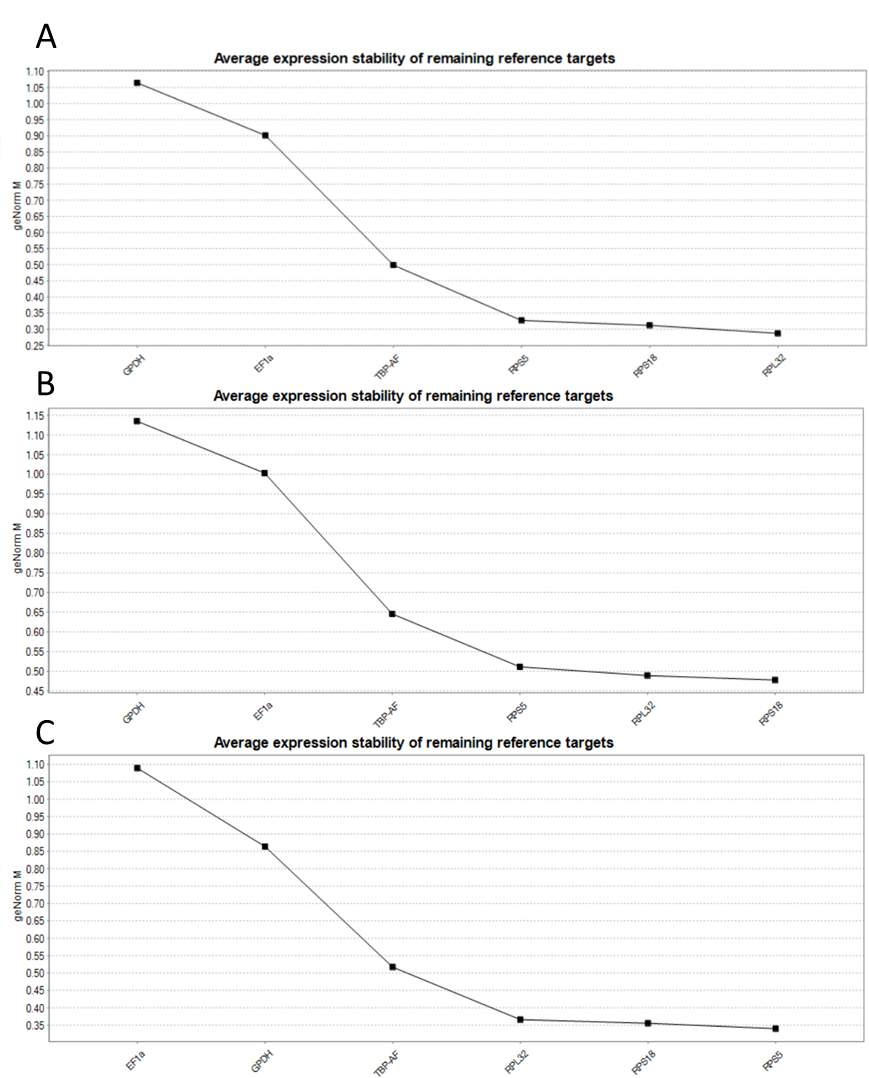


**Figure S10-** Average M-value of target reference genes in tissues, organs and body parts of (A) *Frieseomelitta varia*, (B) *Melipona quadrifasciata*, (C) *Scaptotrigona bipunctata*, resulted from the analyzes done by GeNorm^PLUS^. In all species the output were: *High reference target stability (average geNorm M ≤ 0.5). This is typically seen when evaluating candidate reference targets on a homogeneous set of samples (e.g. untreated cultured cells, or blood from normal individuals).*

**Table S1 –** Reference gene selection for bee species.

| **Organism (bee species)** | **Samples/Experimental conditions** | **Selected reference genes** | **Best reference genes** | **Reference** |
| --- | --- | --- | --- | --- |
| ***Apis mellifera*** | - Larval and pupal stages - Queen tissues - Juvenile hormone treatment | *act, rp49, ef12, tbp-af* | - *act, tbp-af, rp49* - *act, ef1α, rp49* - *ef1α, tbp-af, act* | Lourenço et al., 2008 |
| ***Apis mellifera*** | Heads of newly-emerged bees infected with the bacteria *Escherichia coli* | *act, αtub, gst1*, gapdh, hmbs, rp49*, rpl13a, rpls18, sdha, ubq, tbp** | *act*  *rps18*  *gapdh* | Scharlaken et al., 2008 |
| ***Apis mellifera*** | Brains of adult worker bees with different ages/ behavior | *rpl32, ef1α, gapdh* | *gapdh* | Reim et al., 2013 |
| ***Apis mellifera*** | Larval stages of workers and queens | *rpl19, rps18, gapdh, loc552572, rpl27a, loc727012, ndufa38, rpl10, pros54, u2af38* | *ndufa8*  *pros54* | Cameron et al., 2013 |
| ***Apis mellifera*** | Heads of nurse and forager bees | *awd, rpl12, loc408353, pontin, rp49*, rpl32 (rp49), rps18, tbp, tub*, gapdh* | *rps18*  *gapdh* | Moon et al., 2018a |
| ***Apis mellifera*** | Abdomens of nurse and forager bees | *rp49*, rpl32 (rp49), rps18, tbp, tub*, gapdh, ace2* | *rpl32*  *rps18*  *gapdh* | Moon et al., 2018b |
| ***Bombus terrestres***  ***Bombus lucorum*** | Tissues (labial gland and fat body) | *ak, ef12, pla2, α-tub, gapdh* | *ak, pla2 (b. terrestres)*  *ef1α, pla2 (b. lucorum)* | Hornóková et al., 2010 |
| ***Bombus terrestres*** | Body parts and tissues of bees infected with the virus IAPV | *ef1α, ppia, rpl23, tbp, ubi* | *ppai*  *rpl23*  *ubi* | Niu et al., 2014 |
| ***Euglossa viridissima*** | Larval, pupal stages and abdomen of adults | *act, argk, argk-nod, ef1α, 14-3-3 epsilon, gst1, itpr, rps18, 28s, tbp* | *tbp*  *14-3-3 epsilon* | Boff et al., 2018 |

*Due to problems in the amplification these genes were not used in further analysis

| **Species** | **Reference Gene** | **Development** | | **Sex** | | **Tissue, Organs and Body Parts** | | **Bacterial Infection** | | **Pesticide exposure** | |
| --- | --- | --- | --- | --- | --- | --- | --- | --- | --- | --- | --- |
|  |  | **GeNorm^PLUS^** | **GeNorm (RefFinder)** | **GeNorm^PLUS^** | **GeNorm (RefFinder)** | **GeNorm^PLUS^** | **GeNorm (RefFinder)** | **GeNorm^PLUS^** | **GeNorm (RefFinder)** | **GeNorm^PLUS^** | **GeNorm (RefFinder)** |
| ***Frieseomelitta varia*** | *EF1-α* | 5 | 5 | 6 | 6 | 5 | 6 | 6 | 6 | 5 | 5 |
|  | *GAPDH* | 6 | 6 | 4 | 4 | 6 | 5 | 5 | 5 | 6 | 6 |
|  | *RPL32* | 1 | 1 | 3 | 2 | 1 | 3 | 2 | 1 | 3 | 3 |
|  | *RPS5* | 4 | 4 | 1 | 1 | 3 | 1 | 3 | 3 | 1 | 1 |
|  | *RPS18* | 2 | 1 | 5 | 5 | 2 | 1 | 1 | 1 | 4 | 4 |
|  | *TBP-AF* | 3 | 3 | 2 | 1 | 4 | 4 | 4 | 4 | 2 | 1 |
| ***Melipona quadrifasciata*** | *EF1-α* | 6 | 6 | 6 | 6 | 5 | 5 | 6 | 6 | 5 | 5 |
|  | *GAPDH* | 5 | 5 | 5 | 5 | 6 | 6 | 5 | 5 | 6 | 6 |
|  | *RPL32* | 1 | 1 | 1 | 1 | 2 | 1 | 3 | 3 | 2 | 1 |
|  | *RPS5* | 3 | 1 | 3 | 3 | 3 | 4 | 4 | 4 | 3 | 3 |
|  | *RPS18* | 2 | 3 | 2 | 1 | 1 | 1 | 1 | 1 | 1 | 1 |
|  | *TBP-AF* | 4 | 4 | 4 | 4 | 4 | 3 | 2 | 1 | 4 | 4 |
| ***Scaptotrigona bipunctata*** | *EF1-α* | 6 | 5 | 6 | 6 | 6 | 6 | 6 | 6 | 5 | 5 |
|  | *GAPDH* | 5 | 6 | 5 | 4 | 5 | 5 | 5 | 4 | 6 | 6 |
|  | *RPL32* | 1 | 1 | 1 | 1 | 3 | 1 | 3 | 1 | 1 | 3 |
|  | *RPS5* | 3 | 3 | 3 | 5 | 1 | 1 | 4 | 5 | 2 | 1 |
|  | *RPS18* | 2 | 1 | 2 | 1 | 2 | 3 | 1 | 1 | 4 | 4 |
|  | *TBP-AF* | 4 | 4 | 4 | 3 | 4 | 4 | 2 | 3 | 3 | 1 |

**Table S2** - Ranking of six candidate reference genes for *Frieseomelitta varia*, *Melipona quadrifasciata* and *Scaptotrigona bipunctata* based on their expression stability during development, between sexes, in tissues, organs and body parts, and after bacterial injection and pesticide exposure, according to analyses by GeNorm^PLUS^ and GeNorm from RefFinder.

**REFERENCES**

Boff, S., A. Friedel, A. Miertsch, J. J. Quezada-Euàn, R. J. Paxton, H. M. G. Lattorff, 2018 A Scientific Note of Housekeeping Genes for the Primitively Eusocial bee Euglossa viridissima Friese (Apidae: Euglossini). Sociobiology 65:4. http://dx.doi.org/10.13102/sociobiology.v65i4.3428

Cameron R. C., E. J. Duncan, and P. K. Dearden, 2013 Biased gene expression in early honeybee larval development. BMC Genomics 14: 903. https://doi.org/10.1186/1471-2164-14-903

Horňáková D., P. Matoušková, J. Kindl, I. Valterová, and I. Pichová, 2010 Selection of reference genes for real-time polymerase chain reaction analysis in tissues from Bombus terrestris and Bombus lucorum of different ages. Anal. Biochem. 397: 118–120. https://doi.org/10.1016/j.ab.2009.09.019

Lourenço A. P., A. Mackert, A. S. Cristino, and Z. L. P. Simões, 2008 Validation of reference genes for gene expression studies in the honey bee, Apis mellifera, by quantitative real-time RT-PCR. Apidologie 39: 372–385. https://doi.org/http://dx.doi.org/10.1051/apido:2008015

Moon K., S. H. Lee, and Y. H. Kim, 2018a Evaluation of reference genes for quantitative real-time PCR to investigate seasonal and labor-specific expression profiles of the honey bee abdomen. J. Asia. Pac. Entomol. 21: 1350–1358. https://doi.org/https://doi.org/10.1016/j.aspen.2018.10.014

Moon K., S. H. Lee, and Y. H. Kim, 2018b Validation of quantitative real-time PCR reference genes for the determination of seasonal and labor-specific gene expression profiles in the head of Western honey bee, Apis mellifera. PLoS One 13: e0200369. https://doi.org/10.1371/journal.pone.0200369

Niu J., K. Cappelle, J. R. de Miranda, G. Smagghe, and I. Meeus, 2014 Analysis of reference gene stability after Israeli acute paralysis virus infection in bumblebees *Bombus terrestris*. J. Invertebr. Pathol. 115: 76–79. https://doi.org/10.1016/j.jip.2013.10.011

Reim T., M. Thamm, D. Rolke, W. Blenau, and R. Scheiner, 2013 Suitability of three common reference genes for quantitative real-time PCR in honey bees. Apidologie 44: 342–350. https://doi.org/10.1007/s13592-012-0184-3

Scharlaken B., D. C. de Graaf, K. Goossens, M. Brunain, L. J. Peelman, et al., 2008 Reference Gene Selection for Insect Expression Studies Using Quantitative Real-Time PCR: The Head of the Honeybee, *Apis mellifera*, After a Bacterial Challenge. J. Insect Sci. 8: 1–10. https://doi.org/10.1673/031.008.3301
